# Aflatoxin B_1_ Degradation and Detoxification by *Escherichia coli* CG1061 Isolated From Chicken Cecum

**DOI:** 10.3389/fphar.2018.01548

**Published:** 2019-01-17

**Authors:** Lingling Wang, Jun Wu, Zhiwen Liu, Yutao Shi, Jinqiu Liu, Xiaofan Xu, Shuxian Hao, Peiqiang Mu, Fengru Deng, Yiqun Deng

**Affiliations:** ^1^Guangdong Provincial Key Laboratory of Protein Function and Regulation in Agricultural Organisms, College of Life Sciences, South China Agricultural University, Guangzhou, China; ^2^Key Laboratory of Zoonosis of Ministry of Agriculture and Rural Affairs, South China Agricultural University, Guangzhou, China; ^3^South China Sea Fisheries Research Institute, Chinese Academy of Fishery Sciences, Guangzhou, China

**Keywords:** aflatoxin B_1_, *Escherichia coli*, degradation, detoxification, chicken cecum

## Abstract

Aflatoxin B_1_ (AFB_1_) is one of the most hazardous mycotoxins contamination in food and feed products, which leads to hepatocellular carcinoma in humans and animals. In the present study, we isolated and characterized an AFB_1_ degrading bacteria CG1061 from chicken cecum, exhibited an 93.7% AFB_1_ degradation rate by HPLC. 16S rRNA gene sequence analysis and a multiplex PCR experiment demonstrated that CG1061 was a non-pathogenic *Escherichia coli*. The culture supernatant of *E. coli* CG1061 showed an 61.8% degradation rate, whereas the degradation rates produced by the intracellular extracts was only 17.6%, indicating that the active component was constitutively secreted into the extracellular space. The degradation rate decreased from 61.8 to 37.5% when the culture supernatant was treated with 1 mg/mL proteinase K, and remained 51.3% when that treated with 100°C for 20 min. We postulated that AFB_1_ degradation was mediated by heat-resistant proteins. The content of AFB_1_ decreased rapidly when it was incubated with the culture supernatant during the first 24 h. The optimal incubation pH and temperature were pH 8.5 and 55°C respectively. According to the UPLC Q-TOF MS analysis, AFB_1_ was bio-transformed to the product C_16_H_14_O_5_ and other metabolites. Based on the results of *in vitro* experiments on chicken hepatocellular carcinoma (LMH) cells and *in vivo* experiments on mice, we confirmed that CG1061-degraded AFB_1_ are less toxic than the standard AFB_1_. *E. coli* CG1061 isolated from healthy chicken cerum is more likely to colonize the animal gut, which might be an excellent candidate for the detoxification of AFB_1_ in food and feed industry.

## Introduction

Microbiotas presenting in human and animal intestinal tracts with a high population density, and great diversity, play a crucial role in the growth and health of the hosts. In poultry, 1 g wet weight of cecal content may contain 10^11^ bacteria, which contribute to enhanced nutrient absorption, xenobiotic compounds transformation, and improvements in immune function(Worrell et al., 1989; Kollarczik et al., 1994; Hooper et al., 2001). Based on accumulating evidence, gut microbes efficiently transform mycotoxins into less toxic compounds. Bacteria have been isolated from animal intestines and confirmed to detoxify deoxynivalenol and zearalenone (Guan et al., 2009; Yu et al., 2010; Gao et al., 2018). Aflatoxins (AFs) are a group of toxic and carcinogenic fungal metabolites produced mainly by the fungi *Aspergillus flavus* and *Aspergillus parasiticus*. AFs contamination in cereal grains is a serious problem worldwide, particularly in sub-tropical and tropical areas (Guan et al., 2011). Of the AFs, aflatoxin B_1_ (AFB_1_) is the most hazardous member and has been classified as a Group I naturally occurring carcinogen by the International Agency for Research on Cancer [IARC] (1993). In this study, we intend to isolate AFB_1_ degrading bacteria from chicken cecum for the development of microbial or enzyme products for applications in the food and feed industries.

Several strategies have been reported to detoxify AFs, including physical, chemical, and biological methods. Physical methods include absorption, heating and irradiation (Aziz and Moussa, 2002; Arzandeh and Jinap, 2011; Di Gregorio et al., 2014). Chemical methods include ammonization, solvent extraction and oxidation (Lee and Cucullu, 1978; Norred and Morrissey, 1983; Akbas and Ozdemir, 2006; Zhu et al., 2016). These physical and chemical detoxification methods have many limitations, such as nutritional losses, expensive equipment and time consuming. In comparison, biological methods was found to be most efficient and showed the greatest specificity and environmental soundness. *Nocardia corynebacterioides* was the first reported AFB_1_-detoxifying microorganism (Ciegler et al., 1966). Bacterial and fungal AFB_1_ degradation strains have been continuously to be reported in the subsequent decades. The AFB_1_ degrading fungi *Pleurotus ostreatus, Armilleriella tabescens*, and *Trametes versicolor* have been proved to possess a detoxification function (Liu et al., 1998; Motomura et al., 2003; Alberts et al., 2009; Das et al., 2014). Some bacteria isolated from soil, feces, nuts and other environments effectively degrade AFB_1_, such as *Rhodococcus erythropolis, Mycobacterium fluoranthenivorans, Stenotrophomonas maltophilia, Enterobacteriaceae sp., Myxococcus fulvus, Bacillus subtilis*, and *Pseudomonas putida* (Alberts et al., 2006; Guan et al., 2008; El-Deeb et al., 2013; Samuel et al., 2014; Juri et al., 2015). Although many microorganisms have been reported as degraders of AFB_1_, there was little information about the structure of the degraded products, few studies confirmed the toxicity of the degradation products by cytotoxicity assessment on hepatocellular or animal toxicity test. The currently known microbial degradation metabolites of AFB_1_ and their levels of toxicity were summarized by Verheecke et al. (2016).

The objectives of this study were: (1) to isolate new AFB_1_ degrading bacteria from chicken cecum; (2) to investigate the optimal degradation conditions; and (3) to analysis the degraded AFB_1_ products and confirm the toxicity of the degraded AFB_1_ products.

## Materials and Methods

### Ethics Statement

The protocol of the study was approved by Laboratory Animal Ethics committee of South China Agricultural University in China (Permit No. 00181878). All the animal experiments in this research were performed according to the Regulations for the Administration of Affairs Concerning Experimental Animals of Guangdong province, China. The date of approval by the ethics committee was September 9, 2017.

### Chicken Cecal Samples Collection and AFB_1_ Preparation

Eight healthy broiler chickens (about 12 months old) were purchased for cecal collection in markets near South China Agricultural University, Guangzhou, China. The chickens were treated with cervical dislocation for euthanasia. Approximately 5 g of the cecal contents were mixed with 20 mL of sterile Luria-Bertani (LB) medium (10 g of tryptone, 5 g of yeast extract, 10 g of NaCl, in 1 L of distilled water, pH 7.0). The mixture remained static for 10 min and the supernatant were preserved in glycerol at a final concentration of 15% v/v at –80°C until further analysis. AFB_1_ was purchased from Sigma-Aldrich (St. Louis, MO, United States). A stock solution of 250 μg/mL AFB_1_ was dissolved in DMSO and stored at -20°C.

### Analysis of AFB_1_ Degradation by the Cecal Samples

Eight cecal samples were incubated in LB medium containing 2.5 μg/mL AFB_1_ at 37°C for 72 h shaken at 180 rpm in the dark. AFB_1_ was added to sterile LB medium at a final concentration of 2.5 μg/mL and incubated under the same conditions as the control. Cultures were centrifuge at 1.2 × 10^4^rpm for 5 min. The amount of AFB_1_ in the supernatant was detected using a previously published method with slight modifications as mentioned subsequently (Guan et al., 2008). AFB_1_ was extracted three times with chloroform (1:2, v/v). The chloroform extracts were evaporated, and the residue was dissolved in methanol, filtered (Merck Millipore, Germany, 0.22 μm) and then analyzed by high-performance-liquid chromatography (HPLC) using an Agilent 1260 Infinity liquid chromatography system (Agilent Technologies, Germany) equipped with a G1321B FLD detector and ZORBAX SB-C18 column. The mobile phase was composed of a mixture of solvent A (water/acetonitrile/formic acid, 92/5/0.8, v/v/v) and solvent B (methanol/acetonitrile/formic acid, 92/5/0.8, v/v/v), and separation was performed at a flow rate of 1.0 mL/min. The time of analysis was 35 min. The volume of injection was 10 μl. The retention time of AFB_1_ was 27.6 min. The experiments were conducted with three repeats. The AFB_1_ degradation rate was calculated using the following formula:

(1-AFB_1_ peak area of treatment/AFB_1_ peak area of control) × 100%

All the AFB_1_ degradation rate mentioned in this article were calculated using this formula.

### Isolation and Identification of AFB_1_ Degrading Bacteria From the Cecal Samples

Ten microliters of sample with the highest AFB_1_ degradation were serially diluted with sterilized distilled water, spread on an LB plate, and incubated at 37°C for 24 h. Fifty colonies on the plate were picked and inoculated to sterile LB medium at a final concentration of 2.5 μg/mL AFB_1_, and incubated at 37°C for 72 h with 180 rpm in the dark. Sterile LB medium was incubated at same condition as the control. AFB_1_ degradation rate was determined by HPLC which performed as mentioned above. AFB_1_ degrading isolates were selected and purified for further study. Genomic DNA was extracted using agenomic DNA Miniprep Kit (Axygen, China). The 16S rRNA gene was amplified by PCR using universal primers (27F, 5′-AGAGTTTGATCCTGGCTCAG-3′ and 1492R, 5′-GGTTACCTTGTTACGACTT-3′). The sequences were BLAST on the National Center for Biotechnology Information website and the phylogenetic tree was constructed using MEGA 7.0.

### Detection of the *E. coli* CG1061 Virulence Gene

In an attempt to identify the virulence gene of our isolate *E. coli* CG1061, a multiplex PCR was developed to detection 10 virulence genes (*ipaH, aatA, eltA, vtx2, eae, aggR, vtx1, aaiC, estA*-porcine, and *estA*-human) with a Diarrhoeagenic *E. coli* PCR Kit (Statens Serum Institut, Denmark) which were widely used for detection the virulence of *E. coli*. The details description of these virulence genes has been reported (Persson et al., 2007). Amplification conditions were 94°C for 6 min, followed by 35 cycles of 94°C for 50 s, 57°C for 40 s and 72°C for 50 s, and finally 72°C for 3 min. Amplicons were analyzed by electrophoresis on 1.5% (w/v) agarose gels under standard conditions.

### AFB_1_ Degradation by Intracellular Extracts and Culture Supernatant of *E. coli* CG1061

*Escherichia coli* CG1061 was cultivated in LB liquid medium for 48 h at 37°C with shaking at 180 rpm. After centrifugation at 1.2 × 10^4^rpm for 5 min at 4°C, supernatant and pellets of cultures were collected respectively.

#### Degradation by Intracellular Extracts

The pellets of cultures were washed three times with PBS, resuspended and lysed on ice using an ultrasonic cell disintegrator (JY92-2D, Xinzhi Instruments, Ningbo, China). The disintegrated cell suspension was centrifuged at 8.0 × 10^3^ rpm for 10 min at 4°C. The supernatant was filtered and incubated with 2.5 μg/mL AFB_1_ at 37°C for 24 h, with shaking at 180 rpm in the dark. PBS containing 2.5 μg/mL AFB_1_ was used as the control.

#### Degradation by Culture Supernatant

The supernatant of cultures were filtered (Merck Millipore, Germany, 0.22 μm) and prepared for next experiments. For the heat treatment, the culture supernatant was dipped into boiling water bath for 20 min. For the proteinase K treatments, culture supernatant was treated with 1 mg/mL proteinase K, incubated at 58°C for 3 h. Then all samples were incubated with 2.5 μg/mL AFB_1_ at 37°C for 24 h, with shaking at 180 rpm in the dark. Sterile LB medium containing 2.5 μg/mL AFB_1_ was used as the control. The experiments were conducted with three repeats.

### Effect of Incubation Time, pH and Temperature on Degradation Rate by the *E. coli* CG1061 Culture Supernatant

#### The Dynamics of AFB_1_ Degradation by the Culture Supernatant Change With Incubation Times

Culture supernatant was incubated for 0, 12, 24, 36, 48, 60, and 72 h at 37°C and a pH of 7.0. Sterile LB medium was incubated in same length of time as their control respectively. The assays were performed in the presence of at 2.5 μg/mL AFB_1_, with shaking at 180 rpm in the dark. The experiments were conducted with three repeats.

#### Effects of pH on the Degradation of AFB_1_ by *E. coli* CG1061 Culture Supernatant

The effects of pH on AFB_1_ degradation were studied by adjusting the pH of the culture supernatant to a pH of 4.3, 4.9, 6.5, 7.4, 8.5, and 8.9 with Na_2_HPO_4_ and KH_2_PO_4_ buffer solutions and then incubating the samples at 37°C for 24 h. Sterile LB medium was incubated in same pH value as their control respectively. All assays were performed at 2.5 μg/mL AFB_1_ in the dark without shaking. The experiments were conducted with three repeats.

#### Effects of Temperature on the Degradation of AFB_1_ by *E. coli* CG1061 Culture Supernatant

The culture supernatant was incubated at 4, 25, 37, 55, and 70°C in a pH 7.0 solution for 24 h to confirm the effects of temperature on AFB_1_ degradation. Sterile LB medium was incubated in same temperature condition as their control respectively. All assays were performed at 2.5 μg/mL AFB_1_ in the dark without shaking. The experiments were conducted with three repeats.

### UPLC Q-TOF MS Analysis of AFB_1_ Degradation Products

*Escherichia coli* CG1061 was cultured in LB medium containing AFB_1_ at a final concentration of 12.5 μg/mL for 72 h at 37°C, with shaking at 180 rpm in the dark. The AFB_1_ degradation products were extracted three times with chloroform (1:2, v/v). The chloroform extracts were evaporated, the residue was dissolved in methanol. After filtered (Merck Millipore, Germany, 0.22 μm), the AFB_1_ degradation products were separated using an Agilent 1290 series ultra-high-performance liquid chromatography (UPLC) system. An Agilent Eclipse Plus C18 column was used for separation (50 × 2.1 mm, 1.8 μm). The inject volume was 2 μl. The mobile phase consisted of acetonitrile (A) and water with 0.2% formic acid (B) and 10 mM ammonium formate at a flow rate of 0.3 mL/min. Mass spectrometry analyses were performed using a quadrupole tandem time-of-flight (Q-TOF) G6450B mass spectrometer system in the positive ion mode. Instrument Parameters: gas temperature 300°C, gas flow 8 l/min, Ion spray voltage 4000 V. Mass spectra were acquired in a full-scan analysis in the range of m/z 50–1000.

### Cytotoxicity Determination of AFB_1_ Degradation Products

The toxicity of CG1061-degraded AFB_1_ and standard AFB_1_ was examined in chicken hepatocellular carcinoma LMH cells using the MTT (3-(4,5-dimethylthiazole-2-yl)-2,5-diphenyltetrazolium bromide) method. LMH cells obtained from the ATCC (Catalog No. CRL-2117). *E. coli* CG1061 was cultured in LB medium containing AFB_1_ at a final concentration of 12.5 μg/mL for 72 h at 37°C, with shaking at 180 r/min in the dark. The supernatant of cultures were filtered and prepared as CG1061-degraded AFB_1_ samples. Sterile LB medium containing 12.5 μg/mL AFB_1_ was incubated under the same conditions to prepare standard AFB_1_ samples. Both samples were diluted with William’s *E* medium containing 10% (w/v) fatal bovine serum (FBS) at final concentrations of 3.75, 2.00, 1.00, 0.50, and 0.25 μg/mL. Sterile LB medium was diluted with William’s *E* medium containing 10% (w/v) FBS as the control. LMH cells were incubated with diluted samples in 96-well plates at 37°C for 48 h in a 5% CO_2_ incubator (Thermo, Forma 370), followed by the addition of MTT and an additional incubation for 3 h. Then the formed crystals were dissolved in DMSO for 30 min. The absorption at 490 nm was recorded using a microplate reader (Bio-Rad, Model-680). The experiments were conducted with five repeats. The percentage of cell viability was calculated using the following formula:

(OD_490_ of sample-OD_490_ of Blank)/(OD_490_ of control - OD_490_ of Blank) × 100%

### Toxicity Determination of AFB_1_ Degradation Products *in vivo* Experiment

Fifteen 1-week-old male nude mice were purchased from Guangdong Medical Experimental Center (China) and were randomly allocated to three groups (*n* = 5). Group I, the control only treated with saline; Group II, treated with 100 μg/kg standard AFB_1_ of body weight (BW); Group III, treated with 100 μg/kg CG1061-degraded AFB_1_ of BW. The chloroform extracts and the residue were dissolved in methanol, then diluted in saline and administrated to mice though intraperitoneal injection every alternative day for 5 weeks. After that, all animals were anesthetized by exposure to 10% chloral hydrate. The liver tissue was separated and homogenized in PBS buffer, then centrifuged at 2.5 × 10^3^ rpm for 10 min at 4°C. The supernatant was collected for measurement of alkaline phosphate (ALP) activities, superoxide dismutase (SOD) activities and reduced glutathione (GSH), which are considered as common biochemical markers (Wei et al., 1993; Vipin et al., 2017). These indices were determined by a microplate reader (Bio-Rad, Model-680) using assay kits provided by Jianchen (Nanjing, China). All the animals were maintained at a temperature of 25°C with a 12 h light/dark cycle and were provided with a standard diet and purified drinking water.

### Statistical Analysis

All experiments were conducted with at least three repeats and the data are presented as the means ± standard deviation (SD). One-Way analysis of variance (ANOVA) was performed for the data of degradation and toxicity experiments using the software package SPSS version 16.0, and minimum significant differences were calculated using Duncan’s multiple range test.

## Results

### Isolation and Identification of AFB_1_-Degrading Bacteria

**FIGURE 1:**
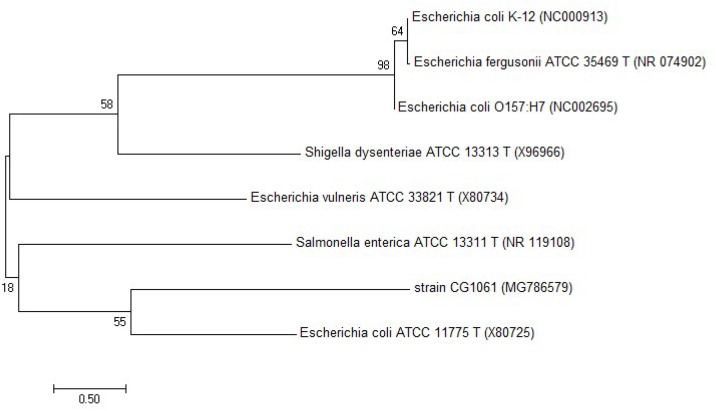
Phylogenetic tree constructed from 16S rRNA gene sequences showing the position of the strain *E. coli* CG1061 using MEGA 7.0. The neighbor-joining method was used and bootstrapped with 1000 replications of each sequence.

**FIGURE 2:**
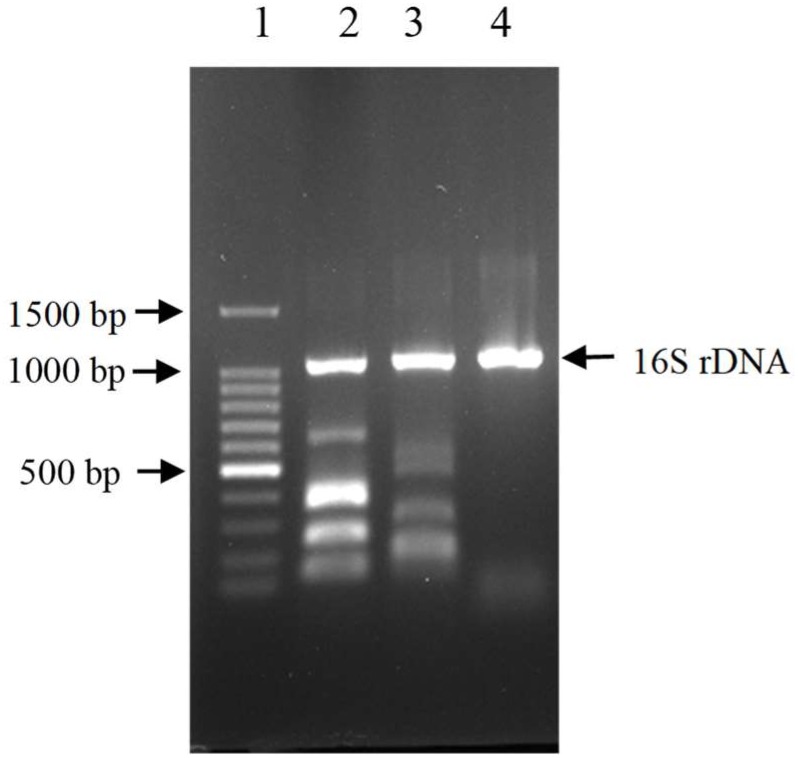
Electrophoretic patterns of simultaneous multi-PCR showing the absence of virulence gene in strain *E. coli* CG1061. Lane 1: 100 bp DNA marker, 1500, 1000, 900, 800, 700, 600, 500, 400, 300, 200, 100 bp (from top to bottom); lane 2 (control 1): 16S rDNA, ipaH, vtx2, eae, vtx1 and estA-porcine (from top to bottom); lane 3 (control 2): 16S rDNA, aatA, eltA, aggR, aaiC, and estA- human (from top to bottom); lane 4: strain CG1061.

The results showed that three out of all eight cecal samples were detected for more than 80% degradation rate. Sample No.8 showed the highest AFB_1_ degradation rate for 93.9%. Fifty colonies picked up from Sample No.8 were detected of AFB_1_ degradation activity by HPLC. The results demonstrated that twelve out of all 50 colonies were detected for more than 80% degradation rate, The left strains had less or no degradation activity at all. Among the isolates, the degradation rate of CG1061 was highest for 93.7% at a final concentration of 2.5 μg/mL AFB_1_ when the cell counts was 10^9^ cfu/mL. It is a gram-negative bacterium, presenting white colonies of approximately 2 mm in diameter on LB plates. The sequence of its 16S rRNA gene (accession number, MG786579 in GenBank) had a 97% similarity with that of *Escherichia coli*. Phylogenetic tree constructed from analysis of 16S rRNA gene sequences showed the relationships of CG1061 and some strains within *Escherichia, Salmonellae*, and *Shigellae* (Figure 1). The phylogenetic position of strain CG1061 was nearest the type strain of *Escherichia coli* ATCC 11775. *E. coli* is the predominant facultative anaerobe in human and animal colonic flora, and covers some pathogenic strains contributing to diarrheal disease (Nataro and Kaper, 1998). In the multiplex PCR (Figure 2), *E. coli* CG1061 produced only the 16S rDNA control band, but not the reported virulence genes. So, CG1061 is a non-pathogenic *E. coli*.

### Degradation Activity of Intracellular Extracts and Culture Supernatant of *E. coli* CG1061

The culture supernatant of the 48 h culture of *E. coli* CG1061 showed an 61.8% degradation rate for AFB_1_, whereas only 17.6% (Table 1) degradation rate was detected for the intracellular extracts, demonstrating that the active component was constitutively secreted into the extracellular space. When the culture supernatant was treated with proteinase K, a significant decrease in degradation rate from 61.8 to 37.5% occurred. After heating of 100°C for 20 min, the degradation rate was 51.3%, indicating that the active components maybe heat-resistant proteins.

**Table 1:** Degradation rates of *E. coli* CG1061 intracellular extracts and culture supernatant.

Component	Initial concentration (μg/mL)	Degradation rates (%)^∗^
Intracellular extracts	2.5	17.56 ± 6.21^d^
Culture supernatant	2.5	61.82 ± 0.23^a^
Culture supernatant with heat-treated	2.5	51.31 ± 6.00^b^
Culture supernatant with proteinase K	2.5	37.52 ± 4.9^c^


*^∗^Different letters among groups means significant different by Duncan’s multiple range test (*P* < 0.05).*

### Effect of Incubation Time, pH and Temperature on Degradation Rate by the Culture Supernatant

**FIGURE 3:**
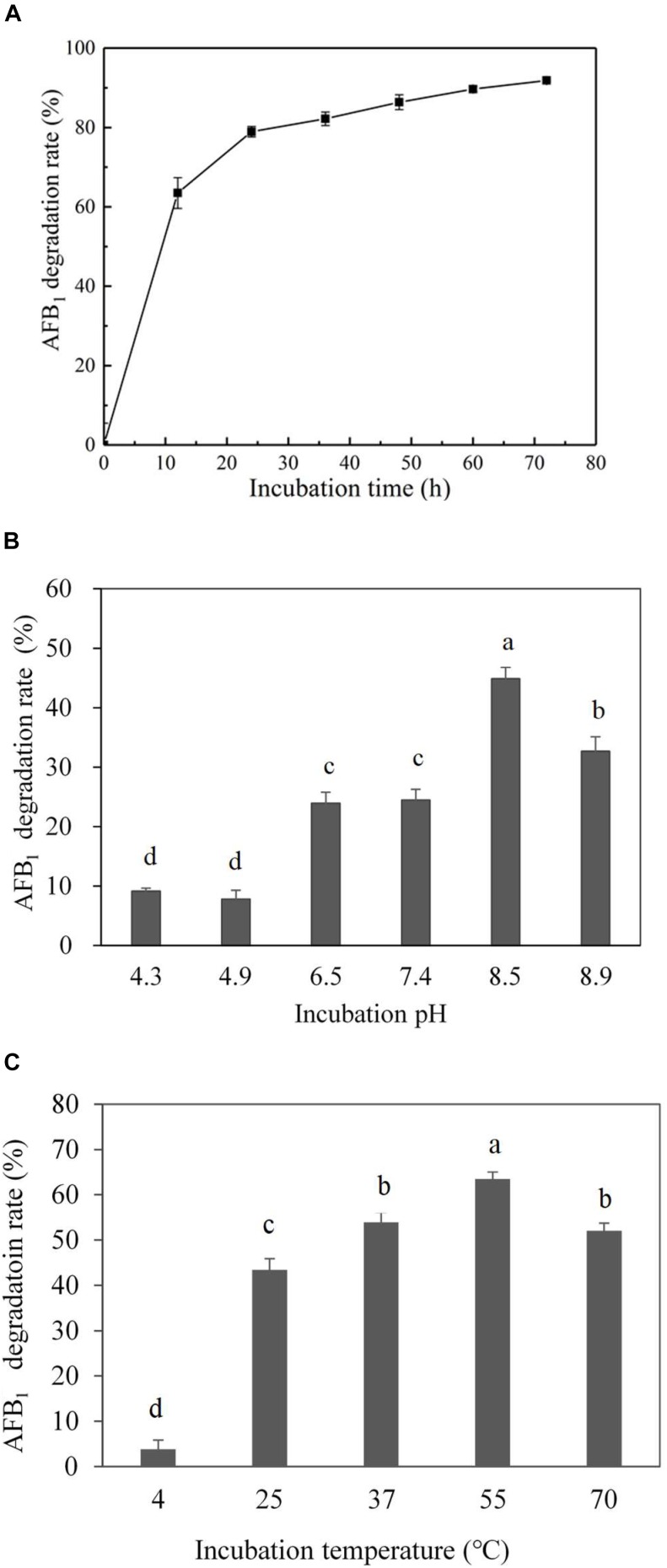
Effect of incubation time, pH and temperature on degradation rate by the culture supernatant of *E. coli* CG1061. **(A)** The dynamics of AFB_1_ degradation by the culture supernatant of *E. coli* CG1061. The experiments were performed in the presence of 2.5 μg/mL AFB_1_ at 37°C, pH 7.0. **(B)** Effects of pH on the degradation of *E. coli* CG1061 culture supernatant. The experiments were performed in the presence of 2.5 μg/mL AFB_1_ at 37°C for 24 h. **(C)** Effects of incubation temperature on the degradation of *E. coli* CG1061 culture supernatant. The experiments were performed in the presence of 2.5 μg/mL AFB_1_ at pH 7.0 for 24 h. Different letters among groups means significant different by Duncan’s multiple range test (*P* < 0.05).

Culture supernatant of *E. coli* CG1061 was incubated with AFB_1_ for 72 h, and AFB_1_ degradation rates were determined every 12 h by HPLC (Figure 3A). In the first 12 h, AFB_1_ degradation rates increased rapidly from 0 to 63.5%, then reach to 79.0% at 24 h, 86.3% at 48 h, and 91.9% at 72 h. The results demonstrated that culture supernatant of *E. coli* CG1061 could significantly reduce AFB_1_ content in first 24 h (reduced nearly 80%). The impact of the incubation pH on the AFB_1_ degradation by the *E. coli* CG1061 culture supernatant was investigated and is shown in Figure 3B. The degradation rates of alkaline conditions were higher than that of acid conditions. The highest degradation rate was 44.8% at pH 8.5. Figure 3C shows the effect of the incubation temperature on AFB_1_ degradation. Over a broad temperature range from 37 to 70°C, the degradation rates were more than 50%. The highest degradation activity appeared at 55°C (degradation rate, 63.5%). The culture supernatant did not lose its degradation activity at 70°C, indicating that the active components involved were thermostable.

### Analysis of Degraded AFB_1_ Products

**FIGURE 4:**
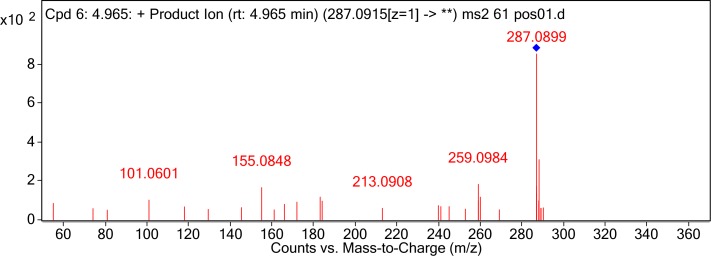
MS/MS spectra analysis of degraded AFB_1_ products. MS/MS spectra of degradation product with 287.09 m/z. Mass spectrometry analyses were performed using a (Q-TOF) mass spectrometer system in the positive ion mode. Mass spectra were acquired in a full-scan analysis in the range of m/z 50–1000.

The degraded products were analyzed by UPLC Q-TOF MS to identify the possible degradation products. The products of AFB_1_ appeared at retention times of 4.965 min and showed the m/z ion peaks at 287.09 for the protonated adduct [M+H] ^+^. It was further analyzed by MS-MS to determine the exact masses of the fragmentation ions (Figure 4). It corresponded to molecular formula C_16_H_14_O_5_. Except this, other possible products of low abundance were also identified as C_8_H_4_O_3_, C_26_H_25_N_3_O_12_S, C_17_H_12_O_7_, and C_17_H_15_O_5_ by the metabolite analysis software MET ID.

### Toxicity of AFB_1_ Degradation Products

**FIGURE 5:**
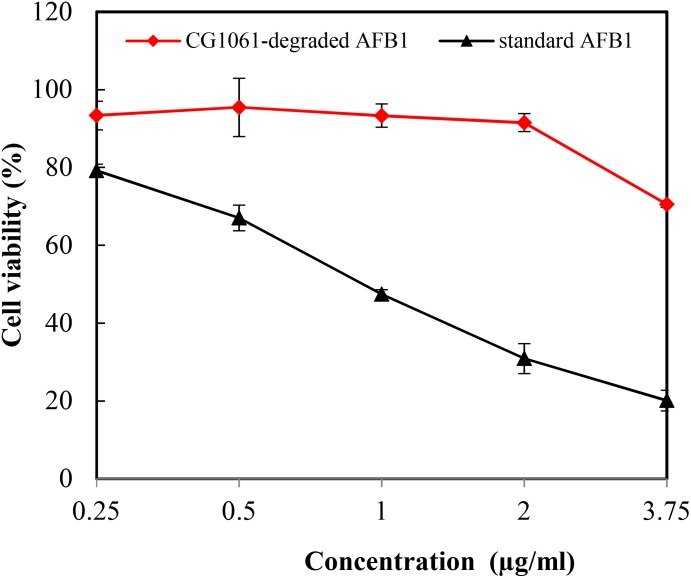
Viability of the LMH cells treated with standard AFB_1_ and with CG1061-degraded AFB_1_. The experiments were performed by the MTT method. LMH cells were incubated with samples in 96-well plates at 37°C for 48 h in a 5% CO_2_ incubator.

In the vitro experiment, CG1061-degraded AFB_1_ and standard AFB_1_ were diluted to final concentrations of 3.75, 2.00, 1.00, 0.50, and 0.25 μg/mL. The viability of LMH cells treated with standard AFB_1_ was from 79.2% (0.25 μg/mL) to 20.1% (3.75 μg/mL), and the cell viability treated with CG1061-degraded AFB_1_ was from 93.4% (0.25 μg/mL) to 70.5% (3.75 μg/mL) (Figure 5). The viability of cells treated with CG1061-degraded AFB_1_ was obviously higher than that of standard AFB_1_ at the same concentration, implying that the cytotoxicity of CG1061-degraded AFB_1_ was much lower than that of standard AFB_1_ on LMH cells.

*In vivo* experiment, after animals were administrated with standard AFB_1_ or CG1061-degraded AFB_1_, the activity of hepatic ALP, SOD, GSH were measured respectively. As shown in Table 2, the activity of hepatic ALP treated with standard AFB_1_ was significant increased (*P* < 0.05) to 148% compared with the control. ALP was an important disease biomarker (Wei et al., 1993), and its significant increase indicated that the administration of AFB_1_ (100 μg/kg of BW) caused liver damage. The activity of ALP did not exhibit any significant differences between that treated with CG1061-degraded AFB_1_ and the control. For the level of SOD and GSH, there is no significant change among all the groups. Based on the results of *in vitro* and *in vivo* experiments, we concluded that AFB_1_ has been transformed into lower toxicity products by *E. coli* CG1061.

**Table 2:** The levels of hepatic ALP, SOD and GSH in nude mice.

Group	ALP (U g^-1^protein)^∗^	SOD (U mg^-1^protein)^∗^	GSH (μM g^-1^protein)^∗^
Group I: control treated with saline	8.96 ± 1.45^a^	3.97 ± 0.15^a^	58.59 ± 1.72^a^
Group II: treated with 100 μg/kg standard AFB_1_ of BW	13.27 ± 0.40^b^	4.01 ± 0.53^a^	58.90 ± 8.61^a^
Group III: treated with 100 μg/kg CG1061-degraded AFB_1_ of BW	8.47 ± 0.71^a^	3.46 ± 0.23^a^	51.10 ± 4.71^a^


*^∗^Different letters among groups means significant different by Duncan’s multiple range test (*P* < 0.05).*

## Discussion

The present study we successfully isolated an AFB_1_ detoxifying bacteria, *Escherichia coli* CG1061 from chicken cecum. *E. coli* CG1061 could eliminate AFs effectively, which reduced AFB_1_ by 93.7% after aerobically cultured for 72 h. The further studies indicated that the active component maybe kinds of heat-resistant compounds, and it is secreted into the extracellular space. In many cases, the supernatant of culture is identified as the degrading matrix and the active component was proved to be heat-resistant. Wang et al. (2017) reported AFB_1_ degradation by the cell-free supernatant of TADC7 microbial consortium was stable up to 90°C. Xia et al. (2017) reported the AFB_1_-degrading activity of isolate *Bacillus subtilis* JSW-1 was predominantly attributed to the cell-free supernatant and this activity was found to be heat stable but sensitive to proteinase K treatment. However, the enzymes responsible for AFB_1_ degradation was not purified and identified, the mechanisms involved are still unknown.

In our research, culture supernatant of CG1061 degraded AFB_1_ for nearly 80% in first 24 h. The results were similar to those reported for *Bacillus subtilis* UTBSP1 (Farzaneh et al., 2012) and *Bacillus licheniformis* CFR1 (Rao et al., 2017). Alberts observed 33.2% of residual AFB_1_ after an incubation in the presence of *Rhodococcus erythropolis* extracellular extracts for 72 h at 30°C (Alberts et al., 2006). *Mycobacterium* DSM44556 degraded more than 90% of AFB_1_ within 4 h at 30°C (Teniola et al., 2005). Wang et al., 2017 reported the degradation rate of a *Fusarium* sp. WCQ3361 cell-free culture in 1 min at 30°C was 70.2%. There were great differences in degradation efficiency from strains to strains. Next, we explored the optimal degradation conditions, the highest degradation activities of culture supernatant of *E. coli* CG1061 appeared at pH 8.5 and 55°C. Xu et al. (2017) purified AF-degrading enzyme from *Bacillus shackletonii* L7, which exhibited the highest activity at pH 8.0 and 70°C. The degradation rate of a *Fusarium* sp. supernatant at 90°C was greater than 73% (Wang et al., 2017), and its optimal reaction pH value was 7.0. Motomura et al., 2003 reported optimum activities of an extracellular enzyme from fungi *Pleurotus ostreatus* at pH values ranging from 4.0 to 5.0 and at the temperature of 25°C. These reports showed the optimal degradation conditions varied with difference microorganisms, suggesting that there may be multiple AFB_1_ metabolic types in microorganisms. Some strains showed high degradation activity at high temperature (more than 70°C), the mechanism of AFB_1_ degradation requires further study.

The enzymes responsible for degradation may have different target sites on AFB_1_ molecule. They were reported for catalyzing the bisfuran ring or coumarin group of AFB_1_. The different action sites lead to different AFB_1_ degradation products. In this study the metabolite C_16_H_14_O_5_ degradated by *E. coli* CG1061 was determined by UPLC Q-TOF MS. Among of reported degradation products of AFB_1_, the molecular formula of AFD_1_ is C_16_H_14_O_5_. AFD_1_ was chemically-transformed from AFB_1_ during the ammonization process (Grove et al., 1984). AFD_1_ is produced by the opening of the lactone ring of AFB_1_. The lactone ring is the actual group of AFB_1_ responsible for its toxic effects (Vanhoutte et al., 2016). Samuel et al. (2014) and Eshelli et al. (2015) reported the bio-transformation of AFB_1_ to AFD_1_ by *Pseudomonas putida* and *Rhodococcus erythropolis* respectively. In this research, whether the product C_16_H_14_O_5_ was AFD_1_ or its isomer remain to be further studied. Liu et al. (1998) reported that *Armillariella tabescense* multienzyme exhibits detoxifying activity by opening the difuran ring of AFB_1_. Besides these, aflatoxicol, AFD_2_ and AFB_2a_ have been reported as AFB_1_ degradation products (Nakazato et al., 1990; Samuel et al., 2014; Chen et al., 2015). In some cases, no degradation products were detected (Alberts et al., 2006; Farzaneh et al., 2012).

AFB_1_ degradation is not always link to less toxic product. So, it is crucial to confirm their toxicity using reliable methods. Approaches of toxicity research of the AFB_1_ metabolites mainly included Ames assay (mutagenicity assessment), SOS-chromotest (mutagenicity assessment), MTT test (cytotoxicity assessment) at present (Alberts et al., 2009; Samuel et al., 2014; Risa et al., 2018). AFB_1_ exposure always leads to liver damage. To determine the toxicity of AFB_1_ degradation products of *E. coli* CG1061, we performed the MTT test on chicken hepatocellular carcinoma cells and the animal toxicity test conducted with mice in this study, which demonstrated the degradation metabolites less toxic than parent AFB_1_. In the animal test, the activity of hepatic ALP was significantly increased (*P* < 0.05) when treated with standard AFB_1_, but for the level of SOD and GSH there was no significant change under same treatment. The results indicated that the activity of hepatic ALP was more sensitive to AFB_1_ than SOD and GSH which were connected with oxidative stress and the antioxidant activity. Kojima and Sakurada (1976) reported ALP activity increased more significantly than that of the other enzymes in the liver of metabolic abnormalities mice.

In summary, non-pathogenic *E. coli* 1061 was reported for effectively removing AFB_1_, its degradation active component was constitutively produced and thermo-stability. The optimal incubation time, pH and temperature were 24 h, pH 8.5 and 55°C respectively. AFB_1_ was bio-transformed to new compounds, which were proved less toxicity by toxicity assessment. From the finding of this investigation, Inoculants of *E. coli* 1061 or enzymes prepared from culture supernatant can be used for detoxification of AFB_1_-contaminated food and feed raw materials.

## Author Contributions

LW designed the experiments and wrote the manuscript. JW analyzed the data and revised the draft. ZL performed the MTT test and animal experiments. JL co-performed the animal experiments. YS detected AFB_1_ degrading rates. XX performed the pathogenicity of strains. SH isolated strains from chicken cecum. PM analyzed the data. FD co-designed the experiments. YD defined the research theme and supervised the conduct of experiments.

## Conflict of Interest Statement

The authors declare that the research was conducted in the absence of any commercial or financial relationships that could be construed as a potential conflict of interest.
